# Identification of a Novel Nucleocytoplasmic Shuttling RNA Helicase of Trypanosomes

**DOI:** 10.1371/journal.pone.0109521

**Published:** 2014-10-14

**Authors:** Alexandre Haruo Inoue, Mariana Serpeloni, Priscila Mazzocchi Hiraiwa, Sueli Fumie Yamada-Ogatta, João Renato Carvalho Muniz, Maria Cristina Machado Motta, Newton Medeiros Vidal, Samuel Goldenberg, Andréa Rodrigues Ávila

**Affiliations:** 1 Instituto Carlos Chagas, FIOCRUZ, Curitiba, Brazil; 2 Departamento de Biologia Celular e Molecular, Universidade Federal do Paraná, Curitiba, Brazil; 3 Departamento de Microbiologia, Universidade Estadual de Londrina, Londrina, Brazil; 4 Instituto de Física de São Carlos, Universidade de São Paulo, São Carlos, Brazil; 5 Departamento de Biologia Celular e Parasitologia, Instituto de Biofísica Carlos Chagas Filho, Universidade Federal do Rio de Janeiro, Rio de Janeiro, Brazil; 6 National Center for Biotechnology Information, National Library of Medicine, National Institutes of Health, Bethesda, Maryland, United States of America; University of Toronto, Canada

## Abstract

Gene expression in trypanosomes is controlled mostly by post-transcriptional pathways. Little is known about the components of mRNA nucleocytoplasmic export routes in these parasites. Comparative genomics has shown that the mRNA transport pathway is the least conserved pathway among eukaryotes. Nonetheless, we identified a RNA helicase (Hel45) that is conserved across eukaryotes and similar to shuttling proteins involved in mRNA export. We used *in* *silico* analysis to predict the structure of *Trypanosoma cruzi* Hel45, including the N-terminal domain and the C-terminal domain, and our findings suggest that this RNA helicase can form complexes with mRNA. Hel45 was present in both nucleus and cytoplasm. Electron microscopy showed that Hel45 is clustered close to the cytoplasmic side of nuclear pore complexes, and is also present in the nucleus where it is associated with peripheral compact chromatin. Deletion of a predicted Nuclear Export Signal motif led to the accumulation of Hel45ΔNES in the nucleus, indicating that Hel45 shuttles between the nucleus and the cytoplasm. This transport was dependent on active transcription but did not depend on the exportin Crm1. Knockdown of Mex67 in *T. brucei* caused the nuclear accumulation of the *T. brucei* ortholog of Hel45. Indeed, Hel45 is present in mRNA ribonucleoprotein complexes that are not associated with polysomes. It is still necessary to confirm the precise function of Hel45. However, this RNA helicase is associated with mRNA metabolism and its nucleocytoplasmic shuttling is dependent on an mRNA export route involving Mex67 receptor.

## Introduction

Chagas disease is a neglected disease endemic to Latin America, where about eight million people are affected [1]. This disease is caused by infection with *Trypanosoma cruzi* (*T. cruzi*). In addition to their medical importance, trypanosomatids are interesting experimental models because the regulation of gene expression in these organisms have some unusual features. Several genes are grouped together under the control of a single promoter region [2], [3] and give rise to long polycistronic transcripts. These transcripts are processed by *trans*-splicing and polyadenylation to form monocistronic messenger RNA (mRNA) [4], [5], [6]. The resulting mature mRNAs are then transported from the nucleus to the cytoplasm, where protein synthesis occurs. Gene expression is controlled mostly by post-transcriptional events and the mechanisms controlling mRNA processing [4], [5], [6] and stability [7], [8], [9] are becoming increasingly understood. However, little is known about the mechanisms of mRNA nucleocytoplasmic transport in these parasites, and the identity of factors that determine the fate of mRNA in the cytoplasm remains to be unveiled.

In yeast and mammalian cells, the bidirectional translocation of macromolecules between the nucleus and cytoplasm (e.g. RNAs to the cytoplasm and transcription factors to the nucleus) involves the nuclear pore complex (NPC), which is composed largely of nucleoporins [10]. The NPC mediates the transport of molecules by interacting transiently with proteins from the β-karyopherin family [11], which are conserved nuclear receptors known as importins and exportins [12]. Crm1 is the major exportin in many organisms [13]. It recognizes a nuclear export sequence (NES) motif in shuttling proteins and its activity is dependent on RanGTP [14], [15]. Only some mRNAs are transported by Crm1 [16], [17], [18], [19]. Instead, most mRNAs are exported by the Mex67 receptor [19], which does not belong to the karyopherin family and functions in a RanGTP-independent manner. During transcription, RNA-binding proteins of the THO complex associate with the nascent mRNA and initiate the formation of the ribonucleoprotein complex (mRNP). This involves the recruitment of processing and export factors, such as Sub2 and Yra1, resulting in the formation of the transcription/export (TREX) complex. The TREX complex interacts with the spliceosome and processed mRNAs are exported through the NPC by the Mex67 receptor and are disassembled in the cytoplasm by the ATP-dependent helicase Dbp5 (DDX19 in humans) [20], [21]. Comparative genomic analyses have demonstrated that the mRNA export pathway is poorly conserved in many parasites, including *T. cruzi* [22], suggesting distinct mechanisms in mammals and parasites.

In this study, we investigated the function of an ATP-dependent DEAD/H RNA helicase (Hel45) in *T. cruzi*. We show that Hel45 shuttles between the nucleus and cytoplasm and is located near to NPCs. We demonstrate that the export of Hel45 is dependent on transcription and we show that it forms ribonucleoprotein complexes not associated with polysomes in the cytoplasm. We also show that Hel45 has a nuclear export signal and its shuttling is dependent on an mRNA export pathway, involving a homolog of the Mex67 nuclear receptor. Our findings also suggest that this protein is involved in mRNA metabolism and its nucleocytoplasmic shuttling is dependent on an mRNA export route involving Mex67 receptor.

## Materials and Methods

### *In silico* analyses

Bioinformatic searches were locally performed using the BLASTP algorithm [23] and *Trypanosoma cruzi* Hel45 (GI: 71418343) as query sequence. Proteome sequences from representative species of different eukaryotic groups were downloaded from the National Center for Biotechnology Information (NCBI) Reference Sequence (RefSeq) database [24]. Analyzed species included: *Saccharomyces cerevisiae* (Fungi), *Homo sapiens* (Metazoa), *Dictyostelium discoideum* (Amoebozoa), *Arabidopsis thaliana* (Plantae), *Plasmodium falciparum* (Chromalveolata), *Toxoplasma gondii* (Chromalveolata), *Trypanosoma cruzi* (Excavata), *Trypanosoma brucei* (Excavata), and *Leishmania major* (Excavata). Multiple sequence alignment of the region (positions 25–365 according to Hel45) comprising the nine diagnostic conserved motifs of DEAD-box helicases were performed using MUSCLE [25]. Identity and similarity percentages were obtained using needle program from the EMBOSS package [26].

Structural homology-based molecular modeling of Hel45 (GeneID 3541696) was carried out by protein searches with the BLASTP of the protein data bank (PDB) database [27]. Alignments of proteins, based on primary and secondary structures, with low levels of sequence identity were generated with the GenTHREADER program [28]. A model was constructed with MODELLER 9v11 [29]. Figures of the structural model were generated with PyMOL software (available at http://www.pymol.org).

The program NESsential [30] (available from http://seq.cbrc.jp/NESsential/) was used for the prediction of classical nuclear export signal (NES) and PredictNLS (available from https://rostlab.org/owiki/index.php/PredictNLS) was used for the prediction of nuclear localization signal (NLS) sequences.

### Parasite cultures

*T. cruzi* Dm28c epimastigotes [31] were maintained in axenic culture in liver infusion tryptose (LIT) medium at 28°C. For drug assays, parasites were treated with 500 ng/ml leptomycin B (Sigma-Aldrich) or 50 µg/ml actinomycin D (Sigma-Aldrich) at 28°C.

RNA interference assay was carried out with procyclic forms of *Trypanosoma brucei* Lister 427 29-13 [32]. *T. brucei* were maintained in SDM-79 medium at 28°C supplemented with 10% fetal bovine serum, G418 (15 µg/ml) and hygromycin (50 µg/ml).

### Polyclonal antibody production

The Hel45 open reading frame (ORF) was amplified by PCR with the oligonucleotide primers Hel45F and Hel45R (Table 1). *T. cruzi* Dm28c was used as the DNA template. The PCR product was cloned into the pDONR^TM^221 vector from Gateway technology (Invitrogen) and was then recombined into the pDEST^TM^17 vector (Invitrogen) to produce a his-tagged Hel45 recombinant, according to the manufacturer’s protocol. Production of recombinant protein was induced in *Escherichia coli* BL21 (DE3) by addition of 1 mM IPTG and incubation for 3 h at 37°C. His-tagged Hel45 protein was purified by affinity chromatography on Ni-NTA resin (Qiagen) under denaturing conditions, and was used to inoculate mice to produce polyclonal antibodies (according to De Souza *et al.* (2010) [33]).

**Table 1:** Oligonucleotides used for PCR.

Primers	Sequence	Use
Hel45F	5′ **GGGGACAAGTTTGTACAAAAAAGCAGGCTTC** ATGGGAGACGTCGAGCAAATAG 3′	Hel45 ORF amplification
Hel45R	5′ **GGGGACCACTTTGTACAAGAAAGCTGGGTC** CTAGAACTGGTCCGCAATATTTGCA 3′	Hel45 ORF amplification
NESF	5′ AATTTGAAACTCTCTGCGACGCCCATGCCGTTATCTTCTG 3′	NES deletion
NESR	5′ CAGAAGATAACGGCATGGGCGTCGCAGAGAGTTTCAAATT 3′	NES deletion
Mex67RNAiF	5′ CCCAAGCTTTGTTAAACCCACTGGAAGGC 3	Mex67 RNAi
Mex67RNAiR	5′ CGCGGATCCAACACACGAGTGAAGTTGCG 3′	Mex67 RNAi

Restriction endonuclease sites are underlined and attB recombination sites are shown in bold.

### Immunoblotting

Proteins were separated by gel electrophoresis (SDS-PAGE) and transferred to a nitrocellulose membrane (Hybond C, Amersham Biosciences). The membrane was blocked with 0.1% Tween 20 and 5% milk in phosphate-buffered saline (PBS). Primary antibodies were diluted in blocking solution at the following concentrations: mouse anti-Hel45 (diluted 1∶500), mouse anti-PABP1 (diluted 1∶100); mouse anti-H2A (kindly provided by Gisele Fernanda Assine Picchi, diluted 1∶250); rabbit anti-Protein A (Sigma-Aldrich, diluted 1∶40,000); mouse anti-Mex67 (diluted 1∶50); mouse anti-GAPDH (diluted 1∶500, kindly provided by Flávia S. Pereira de Souza) and anti-S7 (diluted 1∶1,000).

Antibodies were incubated with the membrane for 1 hour. The membrane was then washed three times in 0.1% Tween 20 in PBS. Bound antibodies were detected by the alkaline phosphatase [34] or peroxidase [33] method.

The nuclear and cytoplasmic extracts for cellular fractionation analysis were obtained by hypotonic lysis of epimastigote forms, as described by Roberts *et al.* (1998) [35].

### Light microscopy

The modified p*Tc*GW vector [36] was used to tag the NT with PTP [37]. The oligonucleotides used to clone the Hel45 ORF are shown in Table 1. The nuclear export signal was deleted by fusion PCR. For this, two fragments of Hel45 were amplified by PCR with the Hel45F/NESR and NESF/Hel45R oligonucleotides (Table 1) and these two amplicons were mixed prior to another round of PCR. The fragment obtained was sequenced and inserted into the p*Tc*GW vector to create a Hel45ΔNES mutant tagged at its N-terminal end with PTP.

*T. cruzi* epimastigotes were transfected with these plasmids, as described by Lu *et al*. (1991) [38]. Stable lines were selected by adding 500 µg/ml G418 to the culture medium. The endogenous and PTP-tagged Hel45 proteins were localized by indirect immunofluorescence assays, as described by Serpeloni *et al.* (2011) [39]. Mouse anti-Hel45 polyclonal antibodies (1∶100 dilution) or rabbit anti-protein A (ProtA) antibodies (1∶40,000 dilution) were incubated with the parasites for 1 hour at 37°C. The parasites were then washed with PBS and incubated with Alexa Fluor 488-conjugated goat anti-mouse IgG, Alexa Fluor 633-conjugated rabbit anti-mouse IgG or Alexa Fluor 594-conjugated goat anti-rabbit IgG antibodies (Invitrogen, 1∶600 dilution), as appropriate, for 1 hour. DNA was stained by incubation with 5 µg/ml DAPI for 15 minutes. Slides were analyzed by fluorescence microscopy (Nikon E600) and images were captured with a CoolSnap PROcf (Media Cybernetics) camera and were analyzed with Image Pro-Plus v. 4.5.1.22 (Media Cybernetics). Images were also obtained by inverted microscopy (Leica DMI6000B) associated with deconvolution software Leica AF6000 (microscope facility RPT07C PDTIS/Carlos Chagas Institute - Fiocruz Paraná).

### Ultrastructural microscopy

Ultrastructural immunocytochemistry of Hel45 in *T. cruzi* epimastigote forms were performed as described by Motta *et al*. (2003) [40]. Samples were blocked for 30 minutes with 3% BSA, 0.5% teleostean gelatin, and 0.02% Tween 20 in PBS pH 8.0, and were then incubated with anti-Hel45 antiserum (1∶50 dilution) for 1 hour. The parasites, on grids, were treated for 30 minutes with blocking solution and were incubated with 10 nm gold-labeled anti-mouse IgG (Sigma-Aldrich) diluted 1∶250 in blocking solution. The grids were washed in blocking solution, stained with uranyl acetate and lead citrate, and were observed with a Zeiss EM-900 transmission electron microscope.

### Mex67 RNAi interference

The ortholog of Mex67 in *T. brucei* was named TbMex67 (GeneID 3664369). For gene knockdown by RNA interference, the region corresponding to 362–845 of the nucleotide sequence was chosen with the RNAit program [41]. A DNA fragment was amplified with the oligonucleotides Mex67RNAiF (forward) and Mex67RNAiR (reverse) (Table1) for cloning into the p2T7-117 vector [42]. A total of 10 µg insert-containing vector was linearized with NotI enzyme and was transfected into procyclic forms of *T. brucei* 29-13 cell line [32].

Transfected parasites were selected by the addition of 5 µg/ml phleomycin to the medium. RNAi was induced by adding 2 µg/ml tetracycline to log phase parasites, and the knockdown confirmed by western blotting with polyclonal antisera anti-Mex67. Anti-GAPDH was used as a loading control.

### Fluorescence *in* *situ* hybridization (FISH) for the detection of mRNA

For the detection of poly(A)^+^ RNA in *T. brucei* and *T. cruzi*, the parasites were harvested, washed in PBS (pH 7.4), fixed by incubation in 4% paraformaldehyde and were allowed to adhere to poly-L-lysine-coated slides for 10 minutes. The slides were washed in PBS and the parasites were permeabilized by incubation with 0.2 M HCl (diluted in PBS) for 10 minutes. For *T. cruzi*, the cells were incubated with prehybridization buffer (35% formamide, 0.02% BSA in 2X SSC buffer) supplemented with 25 µg/ml tRNA, 1 mg/ml salmon sperm DNA (Sigma-Aldrich) and 40 U/ml RNaseOUT (Invitrogen) for 30 minutes at 37°C. For *T. brucei,* the cells were incubated with prehybridization buffer containing 10X Denhardt’s solution, 1 mM EDTA, 35% formamide in 4X SSC and supplemented with 0.5 µg/ml tRNA and 2 mU/ml RNaseOUT for 30 min at room temperature. Digoxigenin-conjugated oligo(dT) probes (6 ng/µl) were diluted in prehybridization buffer and denatured by heating at 65°C for 3 minutes. Hybridization was performed for 16 hours at 37°C. Probe binding was detected by indirect immunofluorescence analysis with mouse monoclonal anti-digoxigenin antibody (Sigma-Aldrich, 1:300 dilution) and Alexa Fluor 488-conjugated goat anti-mouse IgG secondary antibody (Invitrogen, 1∶600 dilution), as described previously. As a control, 100 µg/ml RNase A was added to the pretreatment buffer before probe hybridization.

### Polysome sedimentation profiles

Polysome sedimentation profiles were obtained by the ultracentrifugation of cytoplasmic extracts (1×10^9^ cells) of epimastigotes on sucrose density gradients. The cells were treated with 100 µg/ml cycloheximide for 10 minutes at 28°C and were harvested by centrifugation at 5,000×*g*. Parasites were washed in cold TKMC buffer (10 mM Tris-HCl pH 7.4; 10 mM MgCl_2_; 300 mM KCl) supplemented with 100 µg/ml cycloheximide. Cell pellet was resuspended in 900 µl TKMC buffer supplemented with 100 µg/ml cycloheximide, 10 µg/ml heparin, 10 µM E-64, 10 mM PMSF and was transferred to a new tube containing 100 µl TKMC buffer to which 10% (v/v) NP-40 and 2 M sucrose were added. The lysate was centrifuged at 16,000×*g* at 4°C for 5 minutes and 500 µl of cleared supernatant was centrifuged on a linear sucrose density gradient from 15 to 55% [43]. For micrococcal nuclease treatment, the supernatant was incubated with 500 U/ml micrococcal nuclease in the presence of 2 mM CaCl_2_ for 30 min, and the reaction was blocked by adding 2.5 mM EGTA.

Parasites were treated with 2 mM puromycin for 1 hour at 28°C and were then washed in cold TKMP buffer (10 mM Tris-HCl pH 7.4, 2 mM MgCl_2_, 500 mM KCl). Cells were centrifuged at 5,000×*g* and were resuspended in 900 µl TKMP buffer supplemented with 10 µg/ml heparin, 10 µM E-64, 10 mM PMSF and 1 mM puromycin. Parasites were lysed with 100 µl TKMP buffer supplemented with 10% (v/v) NP-40 and 2 M sucrose. The cell lysate was centrifuged and 500 µl of the clear supernatant was layered onto a sucrose density gradient (15–55%) prepared with TKMP buffer. All gradients were centrifuged at 192,000×*g* for 2 hours at 4°C and fractions were separated with an ISCO gradient fractionation system. Aliquots of 30 µl of each fraction were collected for western blotting with mouse anti-Hel45 and mouse anti-S7 antibodies.

### mRNP isolation

The cytoplasmic fraction of epimastigotes was obtained by hypotonic lysis, as described by Roberts *et al.* (1998) [35]. Poly(A)^+^ molecules were isolated from the cytoplasmic extract with 1 mg magnetic oligo(dT)-conjugated beads from the PolyATract mRNA Isolation System IV (Promega) according to the manufacturer’s protocol. Cytoplasmic extracts were incubated with the beads for 16 hours at 4°C. The beads were washed three times with hypotonic buffer containing 5 mM 2-mercaptoethanol and 1% NP-40, and bound particles were eluted with 0.2% SDS with heating at 95°C for 5 minutes. As a control, 10 µg/ml RNase A was added to the protein extract before its incubation with beads.

## Results

### Comparative analysis and prediction of the structure of the ATP-dependent DEAD/H RNA helicase (Hel45)

**Figure 1:**
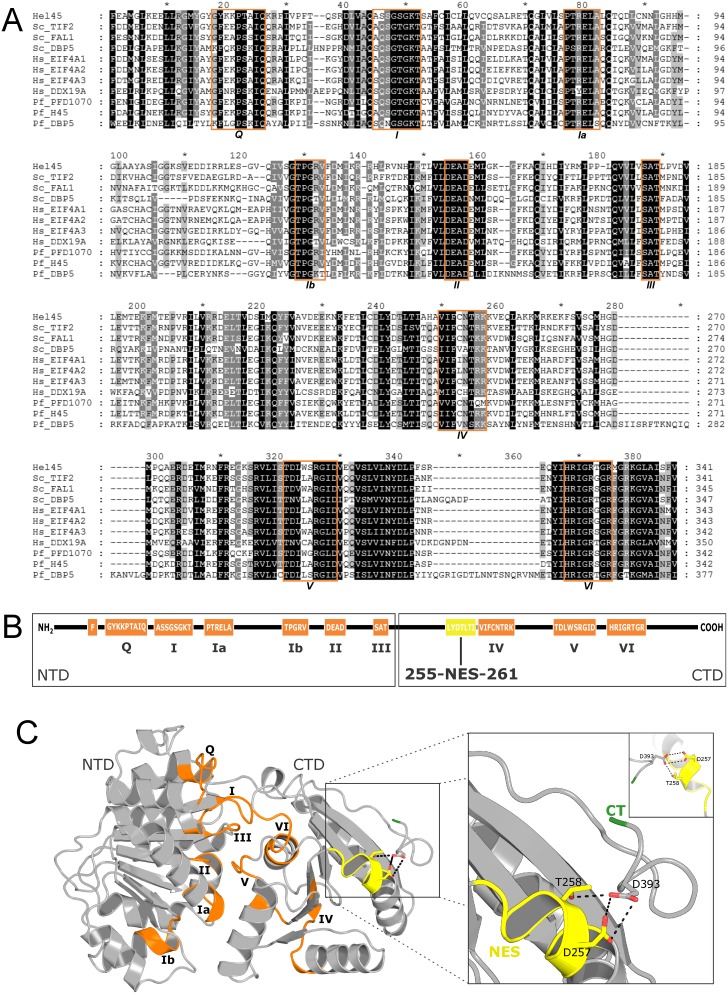
Multiple sequence alignment and prediction of the structure of Hel45. (A) Multiple sequence alignment of the diagnostic conserved region of the DEAD-box helicase family (positions 25–365 according to Hel45). The nine putative conserved motifs (*Q*, *I* (WalkerA), *Ia, Ib, II* (WalkerB), *III, IV, V, VI*) are marked with orange boxes. Alignment columns displaying 100%, more than 90%, and more than 80% of similarity are highlighted in black, dark grey, and light grey, respectively. Sequences are identified with organism abbreviation and gene name, except Hel45. The organism abbreviations are: Sc: *Saccharomyces cerevisiae*, Hs: *Homo sapiens*, Pf: *Plasmodium falciparum*. The sequences have the following GenBank Identifiers (GIs): Hel45 (71418343), Sc_TIF2 (6322323), Sc_FAL1 (398365053), Sc_DBP5 (6324620), Hs_EIF4A1 (4503529), Hs_EIF4A2 (83700235), Hs_EIF4A3 (7661920), Hs_DDX19A (8922886), Pf_PFD1070w (124505577), Pf_H45 (124810293), Pf_DBP5 (6324620). (B) Schematic representation showing the nine conserved helicase motifs are boxed in orange. The N-terminal domain (NTD) contains the motifs Q, I and II for ATP-binding, Ia and Ib for RNA-binding, and III for ATP hydrolysis [44]. The C-terminal domain (CTD) contains the motifs IV and V for RNA-binding, and VI for ATPase and unwinding activities [44]. The predicted nuclear export signal (NES) in the LYDTLTI sequence (255–261 position) is shown in yellow. (C) Molecular modeling of Hel45. The nine motifs are highlighted in orange, the predicted NES (yellow) is close to the CT extremity (green). A zoom of this region (box) shows the side chains of amino-acids D257, T258 and D393, and the interactions that maintain the structure at its C-terminal extremity. The organization of the NES in the CT is shown in the inset (upper right corner).

Previous analysis demonstrated the existence of a *T. cruzi* protein sequence that we named Hel45, conserved across different eukaryotic supergroups examined, including several species of Excavata and Chromalveolata [22]. It is a protein with a predicted molecular weight of 44.9 kDa that belongs to the ATP-dependent DEAD/H RNA helicase family. Comparative analyses by multiple sequence alignment demonstrated that the nine characteristic motifs (*Q*, *I*, *Ia*, *Ib*, *II*, *III*, *IV*, *V*, *VI*) of the DEAD-box helicase protein family [44] were conserved in Hel45 (Figures 1A and 1B). Besides, we observed that Hel45 is similar to shuttling proteins involved in mRNA export routes, as DBP5/DDX19 [45] and the eukaryotic initiation factor 4AIII (eiF4AIII) [46], [47]. This comparative analysis showed that Hel45 is more similar to eiF4A group than to DBP5/DDX19 group of RNA helicases. It had 80.7%, 76.8% and 76.0% of similarity to the eiF4AIII in humans, yeast, and *P. falciparum*, respectively (Table S1). Whereas the similarity with DBP5/DDX19 in humans, yeast and *P. falciparum* was 60.8%, 60.3%, and 52.7%, respectively (Table S1). Hel45 is highly conserved in other trypanosomatids, showing 92.7% and 82.1% of identity, and 97.4% and 91.8% of similarity with *T. brucei* and *L. major*, respectively (Table S1).

We used molecular modeling to predict the structure of Hel45 (Figure 1C) based on structural similarity with related proteins (yeast and human eIF4A; accession numbers in the protein database: 2VSO and 2ZU6, respectively, and human eIF4AIII; accession numbers in the protein database: 2HYI, 2J0S, and 2HXY). We sought to assess the potential role of the protein as an RNA helicase based on this structure. The predicted structure of the presumptive RNA helicase comprised two functional domains (Figure 1C): the N-terminal (NT) domain and the C-terminal (CT) domain. The model of this protein suggested a dynamic spatial conformation of the NT and CT domains, due to a deep cleft between these domains. The two domains were linked by a flexible loop, which is characteristic of proteins with RNA helicase activity.

### Hel45 is present in ribonucleoprotein complexes not associated with polysomes in the cytoplasm

**Figure 2:**
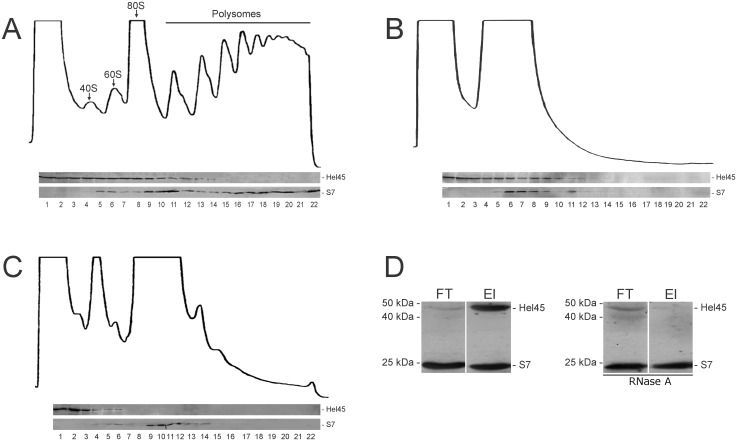
Hel45 is a component of ribonucleoprotein complexes in the cytoplasm. Polysome fractionation by sucrose density gradient. The fractions (1–22) were collected after the sedimentation of cytoplasmic extract from *T. cruzi* treated with 100 µg/ml cycloheximide (A), 2 mM puromycin (B), or 500 U/ml micrococcal nuclease in the presence of 2 mM CaCl_2_ (C). The 40S and 60S ribosomal subunits, the 80S ribosome monomer and polysomes are indicated. A western blot was performed with an anti-Hel45 antibody for each fraction. S7, a small ribosomal subunit protein, was used as a control. (D) mRNP isolation assay. Western-blot analysis with anti-Hel45 and anti-S7 antibodies and mRNPs obtained from the *T. cruzi* cytoplasmic fraction after elution from oligo(dT)-conjugated magnetic beads (El). As a control, cytoplasmic extract was treated with 10 µg/ml RNaseA before mRNP capture. FT = flow-through from cytoplasmic extract not bound to the oligo(dT). El = eluted fraction.

Comparative analysis suggests that Hel45 is very similar to the members of human eukaryotic initiation factor 4A group (eIF4A). We therefore carried out polysome fractionation to assess the potential role of Hel45 in translation. We found that low-density polysome-independent fractions were enriched in Hel45 (Figure 2A). Remarkably, the sedimentation profile of Hel45 on the sucrose density gradient was not modified by treatment with puromycin (Figure 2B), in contrast with that of the ribosomal protein S7 (Figures 2A and 2B). The sedimentation profile of Hel45 was altered only by treatment of the cytoplasmic extract with micrococcal nuclease (Figure 2C). These results suggest that the sedimentation profile of Hel45 is dependent on RNA integrity. Moreover, mRNP precipitation with oligo(dT)-conjugated beads indicated that Hel45 was a component of ribonucleoprotein complexes in the cytoplasm (Figure 2D). These data demonstrate that Hel45 forms mRNPs, but is not associated with polysomes.

### Hel45 is found in both the nucleus and the cytoplasm and clusters around NPCs

**Figure 3:**
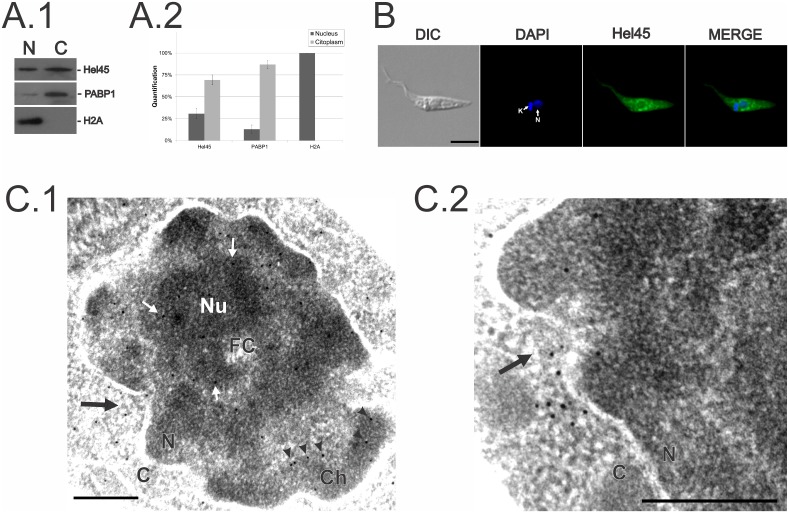
Cellular localization of Hel45 in *Trypanosoma cruzi* epimastigotes. (A.1) Representative results of three independent western blots with cellular extract. N = nuclear extract. C = cytoplasmic extract. PABP1 was used as a cytoplasmic marker and histone H2A was used as a nuclear marker. (A.2) Quantification of western blots by densitometry. Data are expressed as means and standard deviation. (B) Detection of Hel45 by indirect immunofluorescence. DIC = differential interference contrast. DAPI = DNA stained with DAPI. Hel45 =  endogenous Hel45. MERGE = merged DAPI and immunofluorescence images. N = nucleus. K = kinetoplast. Bar = 5 µm. (C.1 and C.2) Ultrastructural microscopy by immunocytochemistry with anti-Hel45 antibodies and 10 nm colloidal gold-coupled anti-mouse IgG. Black arrows = Hel45 labeling on the cytoplasmic side of the NPC. White arrows = Hel45 labeling in perinucleolar regions. Arrowheads = Hel45 labeling in electron-dense chromatin. Nu = nucleolus. FC = nucleolus febrile center. Ch = electron-dense chromatin. N = nuclei. C = cytoplasm. Bar = 0.2 µm.

Western-blot analysis of cellular fractions of *T. cruzi* (Figure 3A.1) showed that about 70% of Hel45 is present in the cytoplasmic fraction and 30% is present in the nuclear fraction (Figure 3A.2). Indirect immunofluorescence assays showed that Hel45 was dispersed throughout the cytoplasm, but showed enrichment around the nucleus (Figure 3B). Ultrastructural immunocytochemical analysis of *T. cruzi* epimastigotes confirmed that Hel45 was present in both the nuclear and cytoplasmic compartments (Figures 3C.1 and 3C.2). In the nucleus, gold particles were present in the periphery of electron-dense chromatin regions and in the periphery of the nucleolus (Figure 3C.1). The protein was dispersed throughout the cytoplasm (not shown) and also accumulated close to NPCs (Figures 3C.1 and 3C.2).

### A predicted nuclear export signal (NES) is essential for Hel45 shuttling between the nucleus and cytoplasm

**Figure 4:**
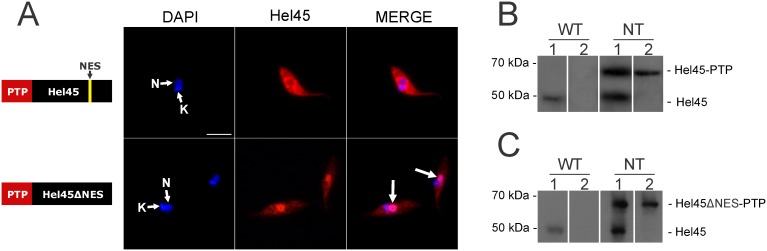
Cellular localization of tagged Hel45 in *Trypanosoma cruzi*. (A) Detection of exogenous Hel45 and a Hel45 NES deletion mutant (Hel45ΔNES) (both tagged with PTP at the NT) by indirect immunofluorescence microscopy with an anti-ProtA antibody. DAPI = DNA stained with DAPI. Hel45 =  localization of tagged Hel45 or Hel45ΔNES. MERGE = merged images for DAPI staining and Hel45 localization. N = nucleus. K = kinetoplast. Arrows = parasites with nuclear accumulation of tagged Hel45. Bar = 5 µm. (B and C) Western blot of total extract from wild-type epimastigotes (WT) and epimastigotes expressing recombinant Hel45 (B) or Hel45ΔNES (C) tagged with a PTP at the N-terminus (NT). Lane 1 =  detection with anti-Hel45 antibodies. Lane 2 =  detection with anti-ProtA antibodies.

The presence of Hel45 in the nucleus and cytoplasm suggests that it acts as a shuttling protein (Figure 3). We therefore searched for nuclear export signals (NES) with the NESsential program, which predicts NES sites on the basis of protein sequence, regional disorder and solvent accessibility criteria [30]. This program recognized a classic nuclear export signal at position 255–261 of the CT region, which consisted of the sequence LYDTLTI. The probability that this putative NES was functional was 63% based on sequence alone. The predicted NES motif is highlighted in Figures 1B and 1C (yellow region). The NES region is located at the end of a helix in the predicted structure (Figure 1C). Structural analysis showed that the side chains of amino-acids D257 and T258 located within the NES motif makes hydrogen-bonds with D393 in the CT region, which keeps the NES motif close to the α-helix (Figure 1C, detailed inset). We addressed the role of this signal by performing fusion PCR to delete the sequence corresponding to the predicted NES (Figure S1). This NES deletion significantly modified the distribution of Hel45, and caused its accumulation in the nucleus (Figure 4A). Western blot analyses confirmed the ectopic expression of tagged Hel45 (Figures 4B and 4C). We also investigated the presence of a conserved nuclear localization signal (NLS) with the PredictNLS program, but no such motif was identified in Hel45 (data not shown).

### The inhibition of transcription blocks Hel45 export to the cytoplasm

**Figure 5:**
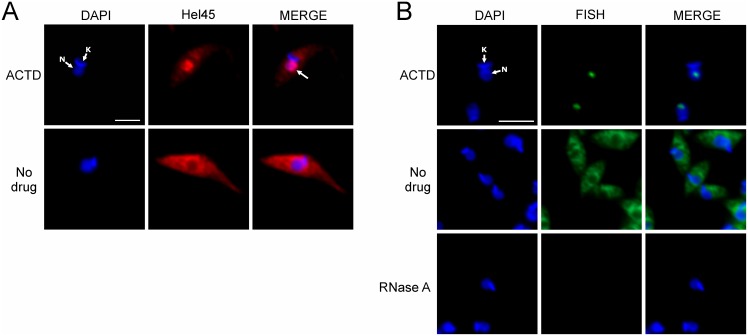
Localization of Hel45 after actinomycin D treatment in *T. cruzi*. Detection of exogenous Hel45 (A) tagged with PTP at the NT by indirect immunofluorescence with an anti-ProtA antibody and of mRNA (B) by fluorescence *in situ* hybridization (FISH) with a digoxigenin-conjugated oligo(dT) probe in *T. cruzi* after treatment with 50 µg/ml actinomycin D (ACTD) for 24 hours. Probe detection was carried out by indirect immunofluorescence with anti-DIG mouse monoclonal antibodies (Sigma-Aldrich, 1∶300 dilution) followed by secondary Alexa Fluor 488-conjugated antibodies (1∶600 dilution). As a control, 100 µg/ml RNase A was incubated with the parasites before probe hybridization (RNase A). DAPI = DNA stained with DAPI. Hel45 =  localization of tagged Hel45. MERGE = merged images for DAPI staining and Hel45 or mRNA localization. N = nucleus. K = kinetoplast. Arrows = parasites with nuclear accumulation of tagged Hel45. Bar = 5 µm.

We found that Hel45 is present in mRNP complexes (Figure 2); therefore, we examined whether blocking the transcription affects Hel45 nucleocytoplasmic export. The treatment of *T. cruzi* with actinomycin D (ACTD) resulted in the accumulation of Hel45 in the nucleus (Figure 5A). For control of actinomycin D activity, it was observed the accumulation of mRNA in the nucleolus (Figure 5B), as previously described [48]. This suggests that Hel45 transport to the cytoplasm is dependent on active transcription.

### Hel45 shuttling is dependent on Mex67 but not on the Crm1 protein

**Figure 6:**
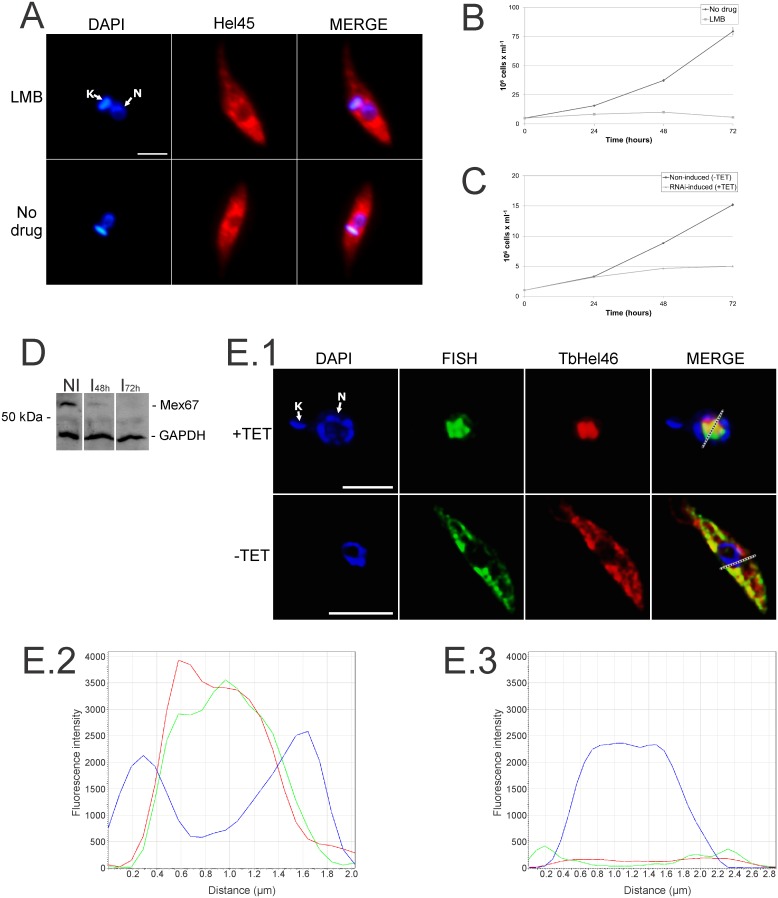
Localization of Hel45 after leptomycin B treatment in *T. cruzi* and localization of the ortholog of Hel45 (TbHel46) in *T. brucei* after Mex67 RNAi induction. (A) Detection of exogenous Hel45 tagged with PTP at the NT by indirect immunofluorescence with an anti-ProtA antibody. *T. cruzi* parasites were treated with 500 ng/ml leptomycin B (LMB) for 24 hours or were untreated (control). (B) Growth curve of *T. cruzi* parasites after treatment with 500 ng/ml leptomycin B (LMB). (C) Growth curve of *T. brucei* parasites after the induction of RNAi against Mex67 with 2 µg/ml tetracycline (RNAi-induced). (B) and (C) represent graphics of a biological replica which the density of cells in the culture was determined by counting in triplicate with a particle counter (Beckman Coulter). (D) Western-blot analysis of total protein extracts from parasites 48 (I_48 h_) or 72 (I_72 h_) hours after the induction of Mex67 RNAi. Non-induced (NI) parasites are shown as a control. The assay was carried out with anti-Mex67 antibodies. Anti-GAPDH antibodies were used as loading control. (E.1) Cellular localization of mRNA by fluorescence *in situ* hybridization (FISH) with a digoxigenin-conjugated oligo(dT) probe and localization of TbHel46 by indirect immunofluorescence Cells were fixed 48 hours after the induction of Mex67 RNAi (+TET). Images were processed by deconvolution software Leica AF6000. DAPI = DNA stained with DAPI. Hel45 =  localization of tagged Hel45. TbHel46 =  endogenous TbHel46 localized with anti-Hel45 antibodies. MERGE = merged images for DAPI staining and Hel45 localization (A) or DAPI staining, FISH and TbHel46 localization (E.1). N = nucleus. K = kinetoplast. (E.2 and E.3) Graphs show the quantification of fluorescence intensity of DAPI (blue), FISH (green), and TbHel46 (red) labelling that was detected across the dotted line in E.1. Fluorescence intensity was plotted in the y-axis for Mex67 RNAi-induced parasites (+TET, Figure E.2) and non-induced parasites (−TET, Figure E.3).

Crm1 is the major exportin in many organisms that recognizes nuclear export sequence (NES) motifs in shuttling proteins [14], [15]. We first investigated if the exportin Crm1 is involved in Hel45 shuttling by treating epimastigotes with leptomycin B (LMB), which specifically inhibits Crm1 activity [49], [50]. LMB did not alter the distribution of Hel45 (Figure 6A) even after treatment with lethal concentrations of the drug, or for long incubation times that affected the growth rate of parasite (Figure 6B). This indicates that Hel45 is not exported in a Crm1-dependent manner.

Mex67 is a nuclear mRNA export receptor in *Trypanosoma brucei* [51], [52]. We hypothesized that shuttling of Hel45 occurs by an mRNA export pathway involving the receptor Mex67, because the export of Hel45 depends on transcription (Figure 5). We tested this hypothesis by knocking down the expression of Mex67 with an inducible system of RNAi in *T. brucei*, because *T. cruzi* does not have a functional RNA interference machinery [53] and lacks an inducible system for gene silencing. The protein orthologous to Hel45 in *T. brucei* showed a predicted molecular weight of 45.51 kDa and we named this protein TbHel46. The amino acid sequence of Hel45 is 92.7% identical and 97.4% similar to that of TbHel46 (Table S1). We used an inducible RNAi system to knockdown the expression of TbMex67, as described previously [52]. The induction successfully prevented the expression of TbMex67 protein (Figure 6D) and impaired the growth of *T. brucei* (Figure 6C). TbMex67 RNAi induction also caused the accumulation of both polyadenylated mRNA (Figures 6E.1 and S2) and TbHel46 (Figures 6E.1 and S3) in the nucleus after 48 hours, which we quantified from the fluorescence intensity of labelling (Figures 6E.2 and 6E.3). This demonstrates that shuttling of TbHel46 depends on the Mex67-mRNA export pathway in trypanosomes.

## Discussion

The mechanisms of molecular exchange between the nucleus and cytoplasm are well characterized in mammals and yeast. However, the proteins and mechanisms involved in the mRNA nucleocytoplasmic transport in several species of parasites are poorly understood. Trypanosomes branched off early from the metazoan lineage, which may account for the conservation of only a few proteins of the mRNA export network in these highly divergent organisms [22]. Regarding to comparative analyses of proteins involved in RNA export, our previous work [22] identified a RNA DEAD-box helicase protein named Hel45. We have considered that Hel45 is conserved across eukaryotes and has similarity to shuttling RNA helicases from mammalian and yeast, like DBP5/DDX19 [45] and eiF4AIII [46], [47]. Then, we decided to investigate the role of Hel45 in mRNA nucleocytoplasmic transport in *Trypanosoma cruzi*.

RNA helicases of the DEAD/H-box family are characterized by the presence of nine conserved motifs that are incorporated into two RecA-like domains. These helicases are involved in several biological steps in RNA metabolism, from transcription to translation [44]. We demonstrate that these nine motifs are conserved in Hel45 and are clustered into the two domains typical of DEAD-box helicases (Figures 1B and 1C). These findings suggest that Hel45 belongs to the RNA helicase family. Comparative analyses of protein sequences from members of eIF4A family and other RNA helicases involved in mRNA metabolism have shown that Hel45 is more similar to eiF4AIII (Figure 1A, Table S1). In metazoan, eiF4AIII is a nuclear protein [54] that associates to mRNA during splicing at region containing the Exon Junction Complex (EJC) and shuttles to the cytoplasm probably to function during Non-sense Mediated Decay (NMD) pathway [46]. However, in trypanosomes no orthologue of eIF4AIII was identified yet as component of EJC core and the role of EJC in trans-splicing remain unclear [55]. Furthermore, it is not clear that a classical NMD exist in trypanosomes [56]. Further investigation is necessary to identify proteins associated to Hel45 before speculating a functional correlation with metazoan eiF4AIII.

Hel45 is present in both cytoplasm and nucleus (Figure 3), and deletion of the predicted NES motif in the CT region resulted in the accumulation of the protein in the nucleus (Figure 4A). These observations confirm that the NES-containing Hel45 is a shuttle protein. The transport of NES-containing cargo is usually mediated by exportins, which interact with domains of inner nucleoporins [57] to mediate transient docking at the NPC [21]. Comparative genomic analysis has shown that Crm1 is the most conserved exportin in diverse organisms [22]. Furthermore, the *T. cruzi* Crm1 contains the CRIME domain, which interacts with RanGTP, and the CCR domain, which is a target of leptomycin B [58]. Surprisingly, we demonstrated that Hel45 nuclear export was not blocked by sustained treatment with leptomycin B (Figure 6A). This indicates that Crm1 is not the receptor involved in transporting Hel45 through the NPC, suggesting that NES is not recognized by Crm1. The NES is essential for Hel45 shuttling, but it may not be the only signal. Indeed, deletion did not result in the complete retention of Hel45 in the nucleus, and some was still present in the cytoplasm (Figure 4A). RNA-binding motifs have also been reported to mediate the nuclear transport of proteins in *T. cruzi* [59]. Therefore we cannot rule out the possibility that the RNA-binding motifs of Hel45 (Figure 1) are also important for transport. Interestingly, only a small number of shuttle proteins have been identified in trypanosomatids and only two RNA-binding proteins identified thus far have NES motifs [7], [60].

Our data indicate that Hel45 is localized at the periphery of dense chromatin domains (Figure 3C.1) called interchromatin granule clusters (IGCs) [39], thought to correspond to regions of active transcription and splicing. Many studies have shown that nascent mRNAs move into these interchromatin spaces [61], [62]. It is likely that an interaction with mRNA is also essential for Hel45 shuttling because Hel45 accumulated in the nucleus when transcription was blocked by actinomycin D (Figure 5). Therefore, Hel45 appears to interact with mRNA during transcription and is transported through the NPC by a specific receptor.

Exportin activity is dependent on RanGTP and, apart from Crm1, other exportins are not conserved in *T. cruzi* [22]. Treatment with leptomicyn B did not block the shuttling of Hel45; therefore, Hel45 may be exported by a RanGTP-independent pathway. Mex67 functions as the receptor for a RanGTP-independent mRNA export pathway in several eukaryotic species. This receptor also mediates mRNA export in *T. brucei*, because knockdown of Mex67 expression leads to accumulation of mRNA in the nucleus [51], [52]. We found that knockdown of Mex67 in *T. brucei* caused an accumulation of the *T. brucei* ortholog of Hel45 in the nucleus (Figure 6E.1). This suggests that Hel45 shuttling is dependent on the Mex67 pathway in trypanosomes.

Even if the molecular modeling has shown structural similarity with members of eIF4A family, Hel45 does not seem to function as a translational factor, because the inhibition of translation did not change the sedimentation profile of Hel45 on sucrose density gradients (Figure 2B). Instead, the sedimentation profile of Hel45 was affected by the treatment of the protein extract with a nuclease (Figure 2C). In addition, Hel45 was present in mRNPs not associated with polysomes (Figure 2D). These results are consistent with previous findings that identified Hel45 as a component of polysome-independent mRNP complexes [43]. Based on these findings, we suggest that Hel45 also interacts with mRNA in the cytoplasm and it is not associated with polysomes. Besides, members of eIF4A family have been shown to possess rather diverse roles in the mRNA lifecycle, although they are highly similar. Their specific and diverse functions are often regulated and dictated by interacting partner proteins [63].

Taking together, our findings suggest that the nucleocytoplasmic shuttling of Hel45 is dependent on a Mex67 mRNA export pathway. However, additional studies are required to assess the precise function of Hel45 in mRNA metabolism. Previous work, indicate that components of the mRNA export pathway in parasites, such as Mex67 [51], [52], present distinct features. This means that the function of specific components needs to be dissected within the context of these particular organisms. Lastly, most factors that play a role in post-transcriptional regulation in parasites are cytoplasmic proteins; therefore, we believe that the identification of nucleocytoplasmic shuttling proteins will improve the knowledge of the factors involved in post-transcriptional regulation of gene expression in parasites.

## Supporting Information

Figure S1 **Deletion of the predicted NES of the Hel45 gene.** Alignment of the Hel45 gene and Hel45ΔNES sequences, obtained with Clustal W2 software. Hel45ΔNES was sequenced and the deletion of NES was confirmed. (N) Nucleotide sequence. (AA) Deduced amino-acid sequence translated from the nucleotide sequence. Asterisks (*) indicate consensus nucleotide sequence. NES = nuclear export sequence.
(TIF)

Figure S2 **Localization of polyadenylated mRNA after induction of RNAi against Mex67 in** ***T. brucei.*** Cellular localization of mRNA with a digoxigenin-conjugated oligo(dT) probe, by fluorescence *in situ* hybridization (FISH). The probe was detected by indirect immunofluorescence assays with a mouse anti-digoxigenin monoclonal antibody (Sigma-Aldrich, 1∶300 dilution) followed by a secondary Alexa Fluor 488-conjugated antibody. As a control, 100 µg/ml RNase A was incubated with the parasites before probe hybridization (RNase A). DAPI = DNA stained with DAPI. MERGE = merged images for DAPI staining and FISH. N = nucleus. K = kinetoplast. Bar = 5 µm.
(TIF)

Figure S3 **Localization of TbHel46 after the induction of RNAi against Mex67 in** ***T. brucei*** **.** Detection of TbHel46 by indirect immunofluorescence with an anti-Hel45 antibody in cells 48 hours after the induction of RNAi against Mex67 (TET). DAPI = DNA stained with DAPI. TbHel46 = endogenous TbHel46 localized with anti-Hel45 antibodies. MERGE = merged images for DAPI staining and TbHel46 localization. N = nucleus. K = kinetoplast. Arrows = parasites with nuclear accumulation of TbHel46. Bar = 5 µm.
(TIF)

Table S1 Similarity of Hel45 with other eukaryotic RNA helicases. Percentage of similarity (upper diagonal) and identity (lower diagonal) among proteins of the DEAD-box helicase family belonging to representative species of different eukaryotic groups. Hel45 is shown as Tcr_Tc00.1047053506587.40_71418343 and the other sequences are named accordingly: organism abbreviation, gene name and GenBank Identifier (GI) delimitated by underscores. The organism abbreviations are: Sce: *Saccharomyces cerevisiae* (Fungi), Hsa: *Homo sapiens* (Metazoa), Ddi: *Dictyostelium discoideum* (Amoebozoa), Ath: *Arabidopsis thaliana* (Plantae), Pfa: *Plasmodium falciparum* (Chromalveolata), Tgo: *Toxoplasma gondii* (Chromalveolata), Tcr: *Trypanosoma cruzi* (Excavata), Tbr: *Trypanosoma brucei* (Excavata), Lma: *Leishmania major* (Excavata).
(XLS)
