# Supplementary figures and images for: Identification of a Novel Nucleocytoplasmic Shuttling RNA Helicase of Trypanosomes
Source: PLoS One. 2014 Oct 14;9(10):e109521. doi: 10.1371/journal.pone.0109521 (PMC4196910; doi:10.1371/journal.pone.0109521)

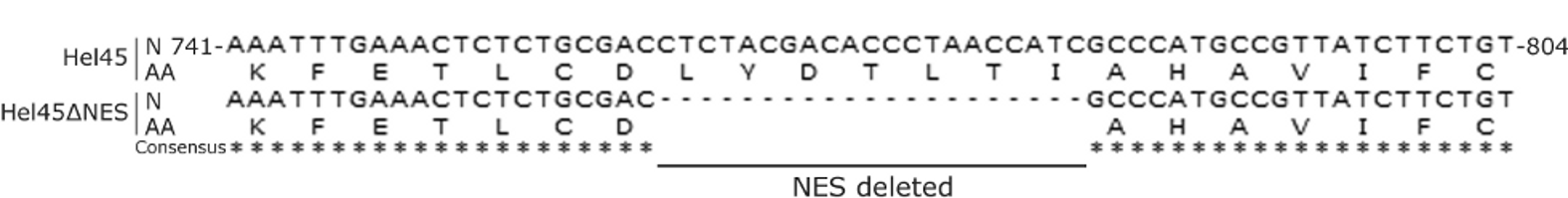

Supplement: Figure S1 — Deletion of the predicted NES of the Hel45 gene. Alignment of the Hel45 gene and Hel45ΔNES sequences, obtained with Clustal W2 software. Hel45ΔNES was sequenced and the deletion of NES was confirmed. (N) Nucleotide sequence. (AA) Deduced amino-acid sequence translated from the nucleotide sequence. Asterisks (*) indicate consensus nucleotide sequence. NES = nuclear export sequence. (TIF) [file pone.0109521.s001.tif]

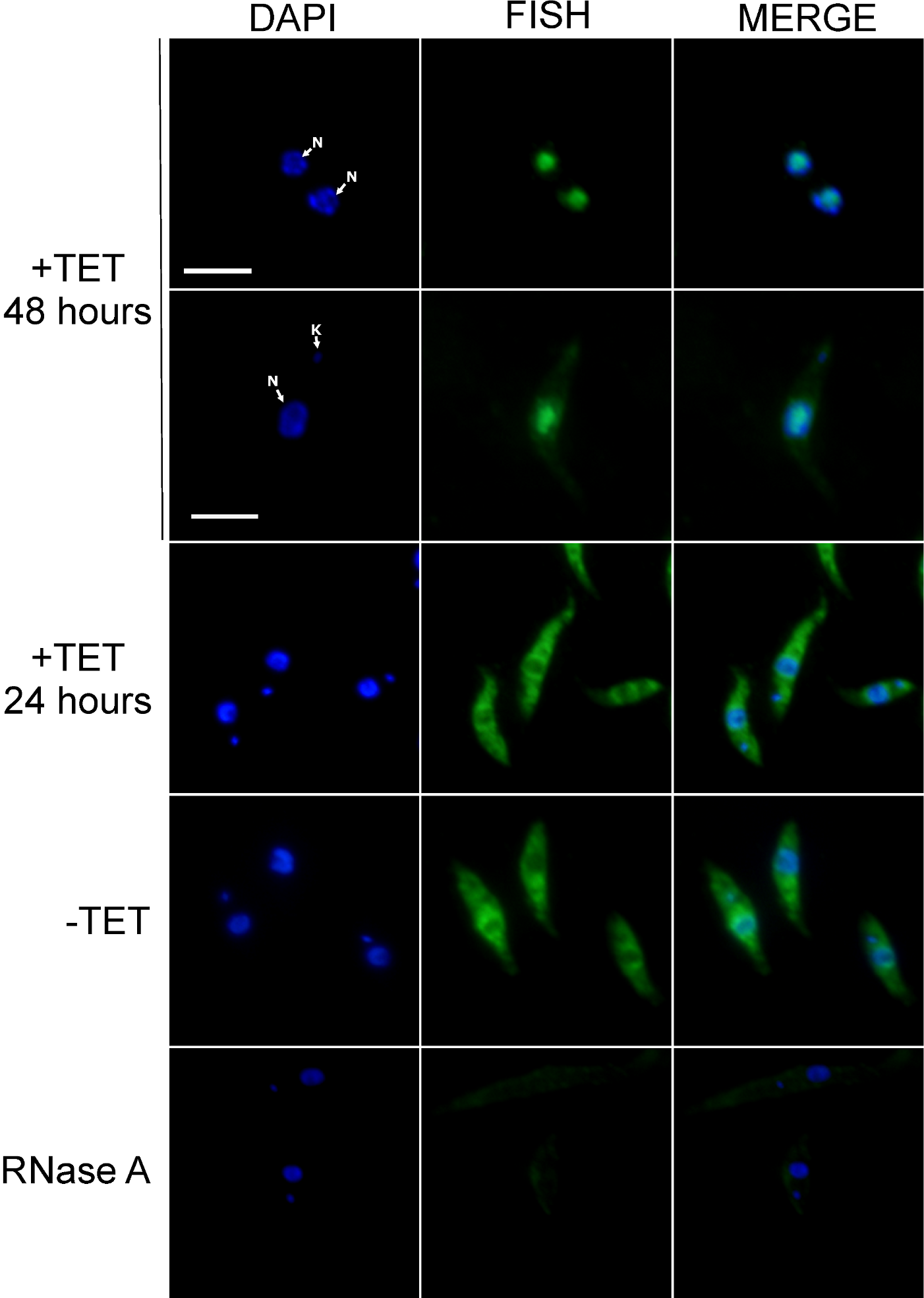

Supplement: Figure S2 — Localization of polyadenylated mRNA after induction of RNAi against Mex67 in T. brucei. Cellular localization of mRNA with a digoxigenin-conjugated oligo(dT) probe, by fluorescence in situ hybridization (FISH). The probe was detected by indirect immunofluorescence assays with a mouse anti-digoxigenin monoclonal antibody (Sigma-Aldrich, 1∶300 dilution) followed by a secondary Alexa Fluor 488-conjugated antibody. As a control, 100 µg/ml RNase A was incubated with the parasites before probe hybridization (RNase A). DAPI = DNA stained with DAPI. MERGE = merged images for DAPI staining and FISH. N = nucleus. K = kinetoplast. Bar = 5 µm. (TIF) [file pone.0109521.s002.tif]

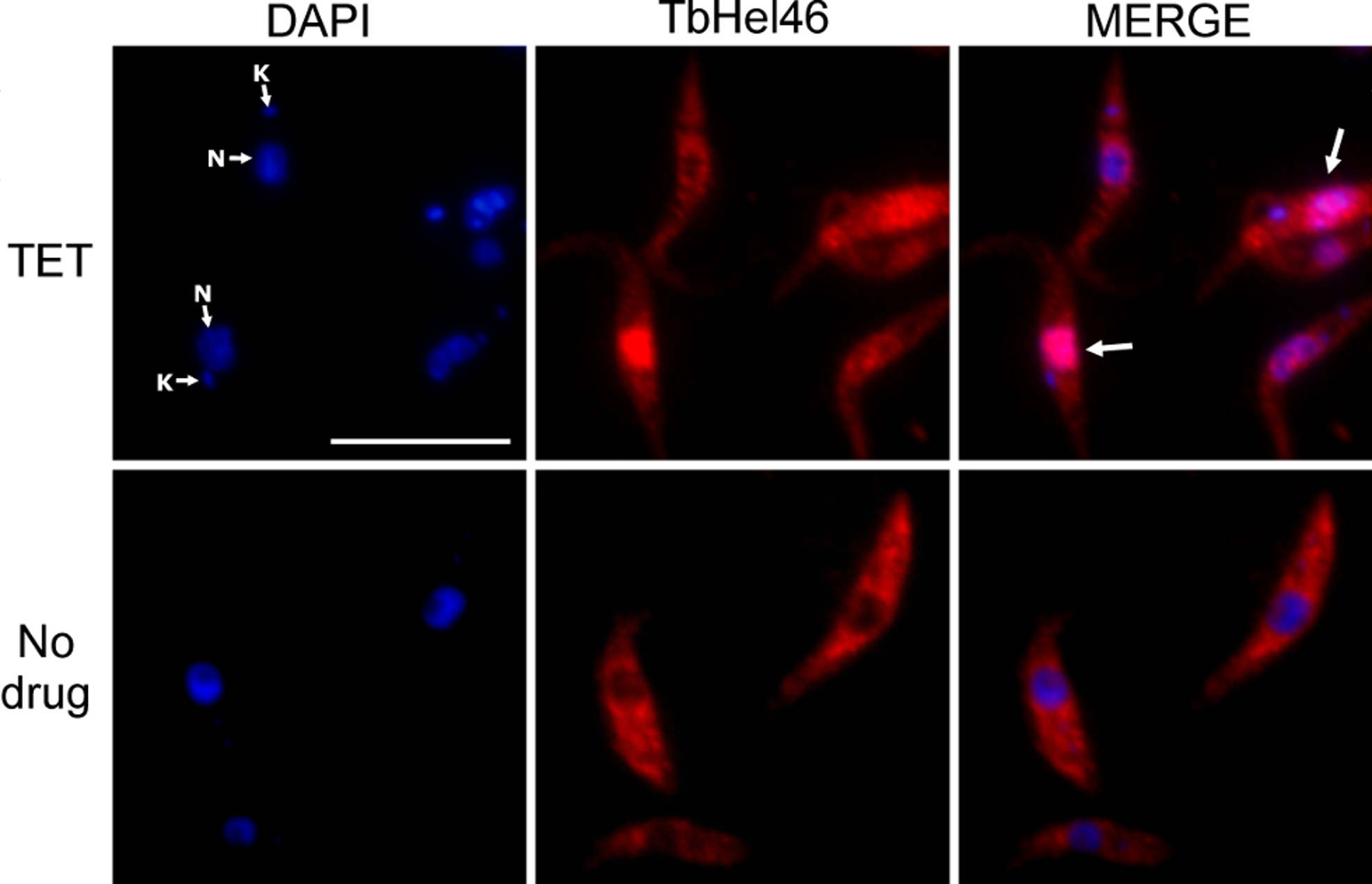

Supplement: Figure S3 — Localization of TbHel46 after the induction of RNAi against Mex67 in T. brucei . Detection of TbHel46 by indirect immunofluorescence with an anti-Hel45 antibody in cells 48 hours after the induction of RNAi against Mex67 (TET). DAPI = DNA stained with DAPI. TbHel46 = endogenous TbHel46 localized with anti-Hel45 antibodies. MERGE = merged images for DAPI staining and TbHel46 localization. N = nucleus. K = kinetoplast. Arrows = parasites with nuclear accumulation of TbHel46. Bar = 5 µm. (TIF) [file pone.0109521.s003.tif]
